# FracFixR: a compositional statistical framework for absolute proportion estimation between fractions in RNA sequencing data

**DOI:** 10.1093/bioinformatics/btaf615

**Published:** 2025-11-20

**Authors:** Alice Cleynen, Agin Ravindran, Nikolay E Shirokikh

**Affiliations:** Institut Montpelliérain Alexander Grothendieck (IMAG), University of Montpellier, Centre National de la Recherche Scientifique (CNRS), Montpellier, Occitanie, 34090, France; International Research Lab France-Australia Mathematical Sciences and Interactions (IRL FAMSI), CNRS International Lab, Mathematical Sciences Institute, Australian National University, Canberra, ACT, 2601, Australia; John Curtin School of Medical Research, Australian National University, Canberra, ACT, 2601, Australia; Institut Montpelliérain Alexander Grothendieck (IMAG), University of Montpellier, Centre National de la Recherche Scientifique (CNRS), Montpellier, Occitanie, 34090, France; Australian Centre for RNA Therapeutics in Cancer (ACRTC) and RNA Innovation Foundry (RIF), School of Human Sciences, The University of Western Australia, Perth, Western Australia, 6009, Australia

## Abstract

**Summary:**

RNA fractionation followed by high-throughput sequencing (RNA-seq) is widely used to study RNA localization, translation, structure, stability and subcellular compartmentalization. Interpreting fractionated RNA-seq data poses a fundamental compositional challenge: library preparation and sequencing depth obscure the original proportions of RNA fractions, which can bias comparisons—particularly when biological changes shift RNA distribution across fractions. This bias compromises comparisons of fraction-specific RNA profiles and limits the utility of standard differential expression methods. Existing approaches using transcript frequency ratios or standard normalization fail to account for the compositional nature of fractionated samples and also cannot estimate the unrecoverable “lost” fraction. We developed FracFixR, a statistical framework that reconstructs original fraction proportions by modeling the compositional relationship between the whole and the fractionated RNA samples. Using non-negative linear regression on carefully selected transcripts, FracFixR estimates global fraction weights, corrects individual transcript frequencies, and quantifies the unrecoverable material. The framework includes methods for differential proportion testing between conditions using binomial GLM, logit, or beta-binomial models. We rigorously validated FracFixR using synthetic data with known ground truth based on naturally observed aligned read distributions and real polysome profiling data from multiple cell lines, demonstrating accurate reconstruction of fraction weights (Pearson correlation >0.85) and enabling detection of differentially translated transcripts between cancer subtypes.

**Availability and Implementation:**

FracFixR is implemented as an R package freely available on GitHub at https://github.com/Arnaroo/FracFixR as well as on the CRAN repository.

## 1 Introduction

Understanding the functional state of a cell often begins with a snapshot of its transcriptome. RNA sequencing (RNA-seq) has become a powerful and widely adopted tool to capture such snapshots, enabling researchers to quantify and compare transcript abundances across different biological conditions (Conesa *et al.* 2016, Wang *et al.* 2009). To gain a more refined understanding of gene expression control, many experimental protocols now include fractionation steps before sequencing. For example, separating cytoplasmic and nuclear RNA can reveal regulatory mechanisms that govern nuclear export and stability of RNA (Christopher *et al.* 2022, Wang *et al.* 2023), while polysome profiling can isolate ribosome-associated transcripts to focus on translated mRNA and identify their ribosomal load (Daro Faye *et al.* 2014, Chassé *et al.* 2017, Horvath *et al.* 2024).

**Figure 1.**
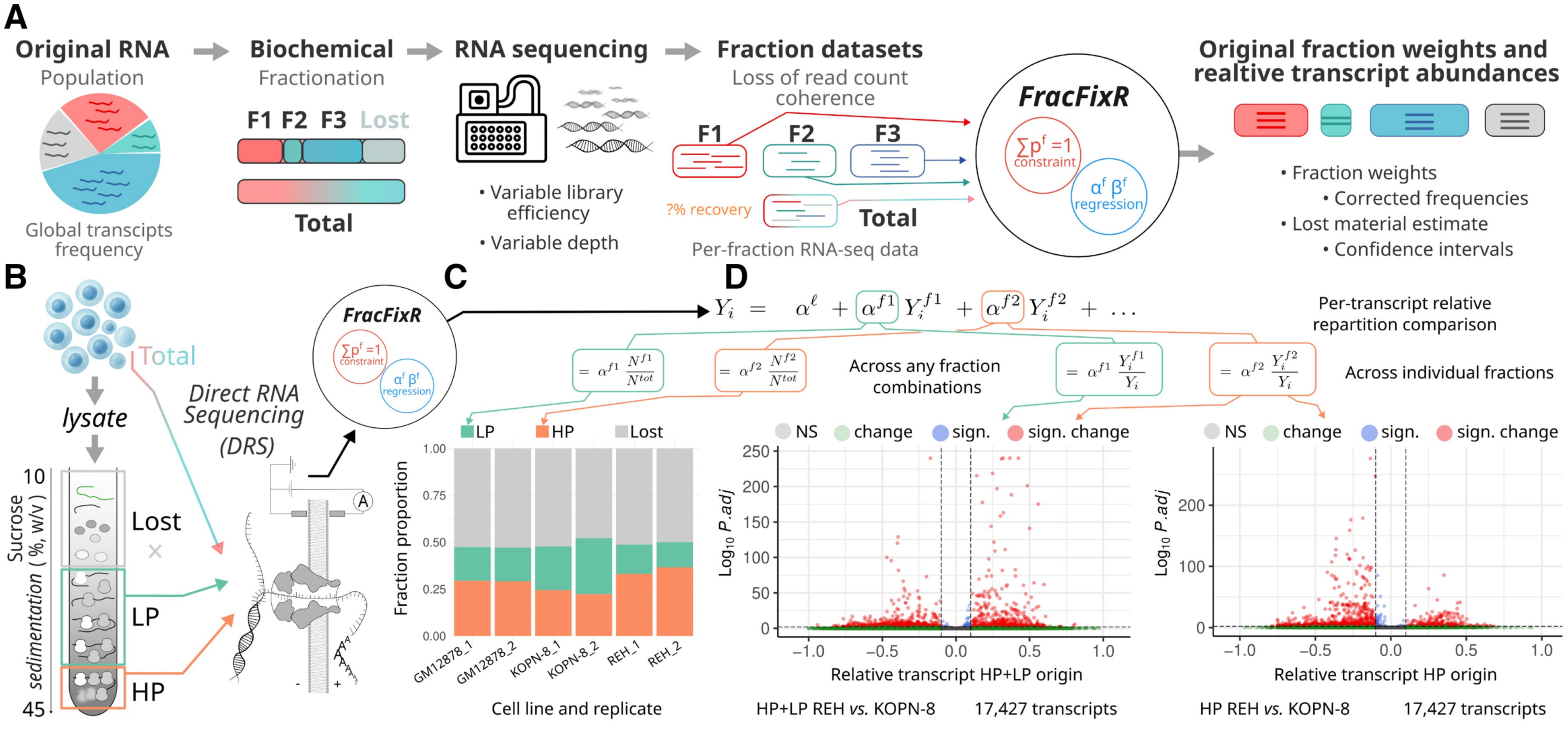
FracFixR: a convenient tool to enable cross-fraction comparisons of relative transcript abundances and recover the original fraction weights in RNA-seq data. (A) FracFixR addresses the different sequencing depth, library efficiency and non-sequenced “lost” material problems of a typical RNA-sequencing experiment of fractionated cell material. (B) Polysome profiling with separation into heavy polysomes (HP) and light polysomes (LP) as a generic RNA fractionation experiment. (C) FracFixR reconstructs polysome profiling fraction weights and calculates the unobserved “lost” fraction in an experiment with differently-relapsing B-ALL cell lines REH and KOPN-8, and a non-malignant B-cell control (GM12878). (D) FracFixR reconstructs individual transcript relative abundance profiles across the fractions and detects many substantial and significant translationally-controlled mRNAs between REH and KOPN8.

However, interpreting fractionated sample RNA sequencing data presents a unique compositional challenge not adequately addressed by standard differential expression analysis methods (Robinson and Oshlack 2010). This occurs because library preparation steps—possibly including amplification of the initial material—can lead to a loss of information about the original fraction proportions (Fig. 1A), especially when the entire sample is not captured in the fractions due to sequencing depth limitations and inability to collect all fractions of the whole. These effects bias and cripple any comparisons of fraction-specific RNA profiles, which often are the major goal of such experiment. The bias may be further inflated by global biological shifts in RNA distribution across the fractions when comparing different conditions (such as stress or drug response), where much RNA can end up in non-recoverable “lost” fraction (Horvath *et al.* 2024, Chassé *et al.* 2017).

Of the common techniques, polysome to monosome ratios (P: M Hedayioglu *et al.* 2022) fails to distinguish the free from translating ribosomes, while transcript frequency-based ratios (such as the Polysome Sedimentation Factor, PSF) can be insufficient because they assume constant global fraction proportions, which is violated when the overall distribution into fractions changes [such as the cell translated overall more or less RNA (Horvath *et al.* 2024)]. Differential transcript abundance (DTA) analysis applied directly across fractions [such as DESeq2 (Love *et al.* 2014)] is inappropriate because it ignores the fact that fractions are parts of a whole. Standard normalization is also inadequate because it assumes proportional scaling, which is violated when sequencing depth (recovery) patterns differ (Bullard *et al.* 2010, Robinson *et al.* 2010).

Yet, RNA localization and compartmentalization are increasingly recognized as fundamental aspects of gene regulation (Moor *et al.* 2017, Holt and Bullock, 2009, Buxbaum *et al.* 2015, Mattick *et al.* 2023), making accurate quantification of RNA distribution across cellular fractions essential for understanding cellular functions.

Here we introduce FracFixR, a general-purpose statistical framework for analyzing RNA-seq data obtained from any fractionation protocol. FracFixR estimates the different fractions contribution to the whole *via* a linear model, which then allows not only to analyze the contribution of a transcript within a given fraction, but also to estimate its relative distribution in each of the fractions (Fig. 1A). FracFixR takes matrix of the mapped read counts as the input and performs compositional fraction normalization. FracFixR outputs: (a) restored weights of original fractions, (b) frequencies of each transcript, and (c) weight of unrecoverable material.

FracFixR can be broadly applied to any RNA fractionation experiment—from subcellular localization studies (Villanueva *et al.* 2024) to ribosome profiling (Ingolia *et al.* 2012, Lareau *et al.* 2014, Shirokikh *et al.* 2017, Archer *et al.* 2016) to RNA-protein complex isolation (Balzarini *et al.* 2023, Urdaneta *et al.* 2019)—enabling researchers to confidently detect biological changes in RNA distribution patterns across diverse experimental systems.

## 2 FracFixR implementation

### 2.1 Development of the FracFixR statistical model

The statistical model is based on a compositional relationship between the whole and the fractions of the RNAs in the cell.

**Notations:** In what follows, we denote by *j* a condition, from which a set of fractions are derived and sequenced. For simplicity, we will denote F the set of all sequenced fractions and its unobserved or “lost” complement, and we will denote *f* an element of F (the superscript *tot* will be dropped when unambiguous). Finally, i∈{1:G} denotes a transcript (or a gene), and ${\tilde{Y_{ij}}}^{f}$ denotes the true (but unobserved) number of transcripts *i* in fraction *f* of sample *j*, while $Y_{ij}{\;}^{f}$ will denote the number of observed counts.

For any given fraction, the sequencing depth factor $s_{j}^{f}=\frac{\sum Y_{ij}{\;}^{f}}{\sum {\tilde{Y_{ij}}}^{f}}$ (or recovery) is assumed to affect all transcripts uniformly, so that $\forall i,EY_{ij}{\;}^{f}=s_{j}^{f}{\tilde{Y_{ij}}}^{f}$.

Furthermore, we introduce $p_{ij}^{f}$ the proportion of transcripts *i* in fraction *f* of condition *j* that originates from fraction *f*, *i.e.* ${\tilde{Y_{ij}}}^{f}=p_{ij}^{f}\tilde{Y_{ij}}$ and are such that $\sum_{f\in F}p_{ij}^{f}=1$, as well as $\mu_{ij}^{f}$ the proportion of total transcripts in fraction *f* of condition *j* that originates from transcript *i*, *i.e.* ${\tilde{Y_{ij}}}^{f}=\mu_{ij}^{f}\sum_{i}{\tilde{Y_{ij}}}^{f}$ and such that $\sum_{i}\mu_{ij}^{f}=1$.

**Model:** From the global relationship in the unobserved full sample; $\tilde{Y_{ij}}=\sum_{f\in {\bar{F}}^{r}}{\tilde{Y_{ij}}}^{f}+{\tilde{Y_{ij}}}^{u}$ we obtain


$$EY_{ij}=\sum_{f\in F}\frac{s_{j}}{s_{j}^{f}}EY_{ij}^{f}$$

and propose to use a non-negative least squares (NNLS) regression to explain the total observed counts by the observed fraction counts, leading to the estimates $\alpha^{f}=\frac{s_{j}}{s_{j}^{f}}$, where $\alpha^{f}$ is the NNLS coefficient for fraction *f*.

**Global fractions:** The relationships between fractions and the total population lead to


$$p_{ij}^{f}\mu_{ij}=\mu_{ij}^{f}\left(\sum_{i}p_{ij}^{f}\mu_{ij}\right),$$

which, combined with the definition of the $s_{j}^{f}$, leads to


$$\frac{s_{j}}{s_{j}^{f}}=\frac{\sum_{i}Y_{ij}}{\sum_{i}Y_{ij}^{f}}\left(\sum_{i}p_{ij}^{f}\mu_{ij}\right),\;\text{and}\;\sum_{i}p_{ij}^{f}\mu_{ij}=\alpha^{f}\frac{\sum Y_{ij}^{f}}{\sum Y_{ij}}.$$

**Individual transcripts:** To estimate individual transcript proportions $p_{ij}^{f}$ in each fraction, we then use the contribution of fraction *f* in the total count $Y_{ij}$  *via* equation


$$p_{ij}^{f}=\frac{\alpha^{f}Y_{ij}^{f}}{\max\left(Y_{ij},\sum_{f}\alpha^{f}Y_{ij}^{f}\right)},\;p_{ij}^{u}=1-\sum_{f}p_{ij}^{f}$$

where the max function ensures that all proportions are positive. To compare true relative abundances of individual transcripts across a given fraction (or set of fractions) between two conditions, we introduce a binomial generalized linear model:


$$\forall j\in \ell ,\;Y_{ij}^{f}|\alpha^{f},Y_{ij}\;∼\mathrm{Bin}(Y_{ij},\alpha^{f}p_{i\ell })$$

and test $H_{0}:p_{i1}^{f}=p_{i2}^{f}$ vs $H_{1}:p_{i1}^{f}\neq p_{i2}^{f}$. P-values are adjusted for multiple testing with the Benjamini-Hochberg procedure Benjamini and Hochberg (1995).

We also provide implementations of a beta-binomial Wald test and a logit-based test as faster alternatives, though they may offer reduced statistical power. Full details of the model and estimation procedures can be found in the Supplementary Materials, section 1.

### 2.2 FracFixR: a convenient and user-friendly R package

FracFixR is implemented as an R package freely available on GitHub. Its standard workflow takes as input a matrix of raw transcript or gene counts from the total population, along with one or more sequenced fractions. It also requires an annotation data frame specifying sample names, experimental conditions, replicates, and fraction types. The package returns an object containing the original data, a matrix of fraction-corrected counts, estimated fraction abundances, normalization coefficients, and diagnostic regression plots (Supplementary Fig. 1).

FracFixR can be installed from GitHub using R command install.packages(“https://github.com/Arnaroo/FracFixR/releses/download/v1.0.0/FracFixR_1.0.0.tar.gz”, repos = NULL, type = “source”). It has modest dependencies, including the nnls package to perform the non-negative linear model, and requires standard data manipulation packages (tidyr, dplyr and matrixStats), and plotting packages (ggplot2, RColorBrewer, EnhancedVolcano). FracFixR makes use of the future.apply package for parallel running of the glm test.

FracFixR is ran using two commands:

- Norma < -FracFixR(CountMatrix, Annotation) runs the NNLS regession and estimates global fractions and individual contributions. The global fraction estimation can be visualized using the command PlotFractions(Norma).
- Results < -DiffPropTest(Norma, Conditions=c(“Condition1”, “Condition2”), Types = “Fraction1”, Test=“GLM”) performs the comparisons between conditions. The resulting volcano plot can be visualized using the command PlotComparison(Results).

Detailed instructions and readme are available in the FracFixR GitHub repository.

## 3 FracFixR performance

In any fractionated RNA-seq experiment, the original ground truth is inherently lost. To rigorously evaluate the performance of FracFixR, we used specialized datasets with either synthetic ground truth or mixtures of real data with known fraction proportions (see Supplementary Materials for details). We then applied FracFixR to real data obtained from long-read direct RNA sequencing (DRS) of the GM12878 Epstein-Barr virus-immortalized normal B-cell line, as well as the B-cell acute lymphoblastic leukemia (B-ALL) REH and KOPN-8 cell lines, to compare their translation efficiency. For each cell line, total RNA was extracted under denaturing lysis conditions, while fractionated RNA was obtained from light and heavy polysomal fractions following sucrose gradient sedimentation (see Supplementary Materials, section 3.2).

### 3.1 FracFixR validation and testing

To construct a ground truth and evaluate modeling accuracy across diverse scenarios, we began with a real count distribution from a DRS experiment containing 1.8 million total reads. We adjusted the total read count to the desired level by subsampling or scaling, then partitioned the data into two observable fractions and a “lost” fraction. The two fractions were allowed to differ in their global representation within the total RNA pool, and transcript abundance profiles within each fraction were varied to reflect distinct biological behaviors. These synthetic datasets were further subsampled to simulate varying recovery rates (the $s_{j}^{f}$ parameters) in both total and fractionated libraries, mimicking different levels of RNA loss and sequencing depth (see Supplementary Figs. 2 and 4). This design captures a range of realistic and challenging conditions, including imbalanced fraction proportions, substantial lost fractions, and uneven recovery or sequencing depth across fractions.

FracFixR consistently and accurately recovered global fraction weights, including the “lost” fraction, across all simulated scenarios, with estimators not depending on the recovery scenario. FracFixR also recovered individual transcript allocations with Pearson correlations near 1, RMSE <0.17, and dispersion <0.15 – even under challenging conditions with asymmetry or data loss (Supplementary Figs. 5–14). The quality of the transcript-level estimators did not depend on the proportion scenario nor recovery of the Total fraction, but significantly improved with sequencing depth of the fractions. This attests to the universality of the model across various data acquisition layouts, and its ability to respond well to an increase of the observation depth As expected, a systematic bias was observed when the fraction weights were mis-estimated, yet the outcome was still closely matching ground truth across all fractions, confirming the statistical validity of the framework.

To understand how the performance expectations could translate to data derived from an actual sequencing experiment, we additionally performed three controlled simulations from raw unmapped fastq reads, mixing RNA-seq of the total cell lysate of REH, KOPN-8 and GM cells, as well as polysomal fractions of REH and KOPN-8 with GM total cell lysate as the “unseen” fraction (Supplementary Fig. 3). The mixing ratios were variable but known. Upon mapping, only the subsets of total mix, REH and KOPN-8 were presented to FracFixR, with GM reads playing the role of a lost “unobserved” fraction. Once again, FracFixR was able to accurately reconstruct the original fraction proportions (weights), as well as provide an estimate of the amount of the “unknown” lost fraction (Supplementary Fig. 15), proving it can handle the biases and variability associated with raw input data.

### 3.2 FracFixR substantially corrects relative transcript abundances in fractionated RNA-seq data

We then applied FracFixR to a representative polysome profiling dataset derived from three cell lines: GM (non-cancerous), REH, and KOPN-8 (both B-ALL subtypes with distinct relapse potential). For each line, we obtained RNA from total lysates as well as from cytoplasmic material fractionated into light (1–3 ribosomes) and heavy (≥4 ribosomes) polysomes (Fig. 1B, Supplementary Materials, section 3.2). As is typical for such experiments, a considerable proportion of RNA was not captured in the sequenced data, due to material loss during lysis and exclusion of non-polysomal fractions.

FracFixR reconstructed the true composition of each sample, including the unsequenced “lost” fraction, and revealed distinct patterns of translation engagement across cell lines. As shown before, a vast majority of transcripts were not associated with ribosomes Jacobson and Peltz [1996], Hedayioglu *et al.* [2022] GM and REH cells showed greater representation of transcripts in heavy polysomes compared to KOPN-8, indicating a higher overall level of engagement with ribosomes (Fig. 1C).

Using FracFixR, we further were able to compare the per-transcript differences between the REH and KOPN-8 cell lines, revealing many transcripts with cell-line specific control. In the combined polysome association analysis enabled by FracFixR , 406 transcripts were more associated with ribosomes in KOPN-8, and 1,335 transcripts were more associated with ribosomes in REH (Fig. 1D, Supplementary Fig. 16). Gene ontology of these hits revealed distinct enriched control pathways. REH GO was enriched for biosynthesis of amino acids, carbon metabolism, glycolysis/gluconeogenesis, spliceosome pathway and nucleocytoplasmic transport, whereas KOPN-8 cells showed a striking enrichment for ribosome components and oxidative phosphorylation. These results suggest a pronounced role of translational regulation in the phenotypic and relapse propensities of these B-ALL subtypes, which is only revealed in these data upon the application of FracFixR.

## 4 Conclusions

FracFixR provides a robust statistical framework for analyzing RNA-seq data from fractionation experiments, addressing the fundamental compositional challenge of recovering true fraction proportions from sequenced libraries. By modeling the relationship between whole and fractionated samples through non-negative linear regression, FracFixR enables accurate estimation of both global fraction weights and individual transcript distributions, including the often-overlooked “lost” fraction. The package’s ability to perform differential proportion testing between conditions makes it convenient and valuable for identifying condition-specific changes in RNA localization or polysome association (Janapala *et al.* 2019, 2021). FracFixR’s implementation as an open-source R package with a straightforward workflow ensures accessibility to the broader research community studying RNA compartmentalization, translation regulation, and subcellular localization. As RNA fractionation techniques continue to evolve and become more sophisticated, FracFixR provides an essential computational tool for extracting meaningful biological insights from these complex datasets while accounting for the inherent biases in library preparation and sequencing depth limitations.

## Supplementary Material

btaf615_Supplementary_Data

## Data Availability

The FracFixR R package is available from GitHub at https://github.com/Arnaroo/FracFixR, and on the CRAN repository at https://doi.org/10.32614/CRAN.package.FracFixR. Synthetic tests data with full ground truth are available at the FracFixR GitHub page and can be flexibly generated using generate_data.py script included in the GitHub repository together with the generation instructions. The replicated DRS data for GM. REH and KOPN8 cell lines are available at NCBI SRA BioProject PRJNA1282458. The global fraction recovery synthetic mixes generated from these data are available as FASTQ and BAM files in the FracFixR GitHub site.
