# Supplementary material for: FracFixR: a compositional statistical framework for absolute proportion estimation between fractions in RNA sequencing data
Source: Bioinformatics. 2025 Nov 20;42(2):btaf615. doi: 10.1093/bioinformatics/btaf615 (PMC12866640; doi:10.1093/bioinformatics/btaf615)
Supplement: btaf615_Supplementary_Data [file btaf615_supplementary_data.pdf]

Supplementary Materials: FracFixR: A compositional statistical framework for absolute proportion estimation between fractions in RNA sequencing data

Alice Cleynen, Agin Ravindran, and Nikolay Shirokikh

Contents

1 FracFixR model . . . 2
1.1 Compositional Relationship (Ground Truth) . . . 2
1.2 Compositional Constraint . . . 3
1.3 Recovery Parameters (Sequencing Depth Effect) . . . 3
1.4 Estimation *via* Non-Negative Least Squares (NNLS) . . . 3
1.5 Connecting Recovery Parameters to α Coefficients . . . 4
1.6 Estimation Outputs . . . 4
1.6.1 Global Fraction Weights . . . 4
1.6.2 Individual Transcript Proportions . . . 5
1.7 Statistical Testing . . . 5
1.7.1 Differential Proportion Analysis . . . 5
1.8 Summary . . . 5
2 FracFixR structure . . . 6
3 Synthetic and Real Data Generation . . . 7
3.1 Fully synthetic data . . . 7
3.2 Materials and Methods for model cell-lines . . . 8
3.2.1 Cell lines and growth conditions . . . 8
3.2.2 Total (whole cell) RNA extraction . . . 8
3.2.3 Translatome capture using polysome profiling . . . 8
3.2.4 Nanopore direct RNA sequencing . . . 11
3.3 Controlled DRS read mixing experiment . . . 11
4 FracFixR results on synthetic data . . . 13
4.1 Fully synthetic data . . . 13
4.2 Controlled real data . . . 20
5 FracFixR analysis of polysome profiling data . . . 21

1 FracFixR model

RNA fractionation experiments separate cellular RNA into compartments (e.g., cytoplasmic vs. nuclear, or ribosome-bound vs. free RNA). A fundamental challenge arises because sequencing depth and library preparation obscure the original proportions of these fractions. FracFixR addresses this compositional problem by modeling the relationship between the whole RNA sample and its fractions.

The core principle: If we sequence both the total RNA pool and its fractions independently, we can mathematically reconstruct the true fraction proportions by exploiting their compositional relationship, even when substantial material is lost or unsequenced.

This document provides the complete mathematical framework underlying FracFixR.

The statistical model is based on a compositional relationship between a whole RNA read set and read sets from fractions of RNA derived from the whole RNA.

Notations: In what follows, we denote:

- $\mathcal{F} = \{u\} \cup \bar{\mathcal{F}} = \{u, \emptyset\} \cup \mathcal{F}^r$ is the set of whole sequenced fraction $\emptyset$, all sequenced fractions $\mathcal{F}^r = \{f\}$, and its unobserved or 'lost' complement u,
- $j \in \{1, n\}$ is a condition from which sequencing will be obtained, both from the whole material and from different fractions,
- $i \in \{1 : G\}$ denotes a unit of read belonging, such as a transcript or a gene,
- $\widetilde{Y_{ij}}^f, f \in \bar{\mathcal{F}}$ denotes the true (but unobserved) number of transcripts i in fraction f of sample j, and
- ${Y_{ij}}^f, f \in \bar{\mathcal{F}}$ will denote the number of observed counts.
- p_{ij}^f is the proportion of transcripts i in fraction f of condition j that originates from fraction f.
- μ_{ij}^f is the proportion of total transcripts in fraction f of condition j that originates from transcript i.

1.1 Compositional Relationship (Ground Truth)

Consider the RNA molecules in a condition of interest (a cell, or a bulk of cells). By definition, the true total RNA equals the sum of all its fractions:

$$\widetilde{Y_{ij}} = \sum_{f \in \bar{\mathcal{F}}^r} \widetilde{Y_{ij}}^f + \widetilde{Y_{ij}}^u \tag{1}$$

Interpretation: The total number of transcript i molecules equals those in all sequenced fractions plus those in the unobserved/lost fraction. Without loss of generality, in the following we will assume that there are two fractions of interest. The results will hold equally true for any number of fractions ≥ 1. Then we can re-write Equation (1)

$$\begin{aligned} \widetilde{Y_{ij}} &= \widetilde{Y_{ij}}^{f_1} + \widetilde{Y_{ij}}^{f_2} + \widetilde{Y_{ij}}^u \\ &= p_{ij}^{f_1}\widetilde{Y_{ij}} + p_{ij}^{f_2}\widetilde{Y_{ij}} + p_{ij}^u\widetilde{Y_{ij}} \end{aligned} \tag{2}$$

where $\forall i, j \ \sum_{f \in \bar{\mathcal{F}}^r} p_{ij}^f + p_{ij}^u = 1$.

Interpretation: Each transcript's total abundance can be decomposed into its proportional contributions from each fraction, and these proportions must sum to 1 (compositional constraint).

1.2 Compositional Constraint

By definition of p_{ij}^f and μ_{ij}^f, we have:

$$\begin{aligned}\widetilde{Y_{ij}}^f &= p_{ij}^f \widetilde{Y_{ij}} = p_{ij}^f \mu_{ij} \sum_i \widetilde{Y_{ij}}, \quad \text{and} \\ \widetilde{Y_{ij}}^f &= \mu_{ij}^f \sum_i \widetilde{Y_{ij}}^f = \mu_{ij}^f \sum_i p_{ij}^f \widetilde{Y_{ij}} \\ &= \mu_{ij}^f \sum_i p_{ij}^f \mu_{ij} \sum_{i'} \widetilde{Y_{i'j}},\end{aligned}$$

. This leads to::

$$p_{ij}^f \mu_{ij} = \mu_{ij}^f \left(\sum_i p_{ij}^f \mu_{ij} \right). \tag{3}$$

Interpretation: This equation establishes a key compositional relationship: the fraction of transcript i in fraction f (left side) must equal the relative abundance of i within that fraction, scaled by the total weight of that fraction (right side). This constraint allows us to connect individual transcript proportions to global fraction weights.

1.3 Recovery Parameters (Sequencing Depth Effect)

A fundamental assumption is that observed counts ${Y_{ij}}^f$ represent the true counts $\widetilde{Y_{ij}}^f$ scaled by a uniform sampling factor (sequencing depth limitation). Thus, for any $f \in \mathcal{F}$, there exists a non-negative parameter s_j^f such that for all i, $\mathbb{E}{Y_{ij}}^f = s_j^f \widetilde{Y_{ij}}^f$. This parameter is called the **recovery** parameter.
Interpretation: Each sequenced library captures only a fraction (s_j^f) of the true molecules present, but this capture rate affects all transcripts uniformly within that library. This is the "sequencing depth factor" or "recovery rate." Thus from Equation (2) we have

$$\mathbb{E}Y_{ij} = \frac{s_j}{s_j^{f_1}} \mathbb{E}Y_{ij}^{f_1} + \frac{s_j}{s_j^{f_2}} \mathbb{E}Y_{ij}^{f_2} + s_j \widetilde{Y_{ij}}^u. \tag{4}$$

Interpretation: The observed total counts can be expressed as a weighted sum of the observed fraction counts, where weights are the ratios of recovery parameters. This is the key insight that allows estimation.

1.4 Estimation *via* Non-Negative Least Squares (NNLS)

We therefore propose to use non-negative least squares (NNLS) regression to estimate these ratios of recoveries:

$$Y_{ij} = \alpha_j^{f_1} Y_{ij}^{f_1} + \alpha_j^{f_2} Y_{ij}^{f_2} + c_j + \varepsilon, \quad \varepsilon \sim \mathcal{N}(0,1),\ c_j, \alpha_j^{f_1}, \alpha_j^{f_2} \geq 0. \tag{5}$$

Note that the Y_{ij} are counts and should be modeled with count distributions, however in practice we will limit the regression to transcripts with the highest quantiles of numbers of reads, leading to large counts that can be approximated by Gaussian distributions.

Rationale:

- We use ordinary least squares because transcripts with high read counts (selected for this regression) can be well-approximated by Gaussian distributions

- Non-negativity constraints ensure biologically meaningful coefficients (proportions cannot be negative)
- The α coefficients estimate the recovery ratios $\frac{s_j}{s_j^f}$
- The intercept c_j captures the unobserved fraction

Note on model choice: While counts should ideally be modeled with count distributions, we restrict the regression to high-quantile transcripts where large counts permit Gaussian approximation, greatly simplifying computation while maintaining accuracy.

1.5 Connecting Recovery Parameters to α Coefficients

From the definitions of recovery parameters:

$$s_j = \frac{\sum_i Y_{ij}}{\sum_i \widetilde{Y_{ij}}} \quad \text{and} \quad s_j^f = \frac{\sum_i Y_{ij}^f}{\left(\sum_i p_{ij}^f \mu_{ij}\right) \sum_{i'} \widetilde{Y_{i'j}}},$$

Therefore:

$$\frac{s_j}{s_j^f} = \frac{\sum_i Y_{ij}}{\sum_i Y_{ij}^f} \left(\sum_i p_{ij}^f \mu_{ij} \right),$$

Combining with Equations (4) and (5), we obtain the estimate::

$$\sum_i p_{ij}^f \mu_{ij} = \alpha^f \frac{\sum Y_{ij}^f}{\sum Y_{ij}} = \alpha^f \frac{N_j^f}{N_j},$$

where α^f is the NNLS coefficient for fraction f, N_j^f is the total reads in fraction f, and N_j is the total reads in the whole sample, and the unobserved fraction is captured by the intercept.

Interpretation: The NNLS coefficient α^f, scaled by the ratio of library sizes, directly estimates the global weight of fraction f in the original sample.

1.6 Estimation Outputs

1.6.1 Global Fraction Weights

The proportion of fraction f in the global population is estimated as:

$$P_j^f = \frac{\sum_i \widetilde{Y_{ij}^f}}{\sum_i \widetilde{Y_{ij}}} = \frac{s_j}{s_j^f} \frac{N_j^f}{N_j} = \alpha_j^f \frac{N_j^f}{N_j}. \tag{6}$$

Interpretation: This gives the true weight of each fraction in the original biological sample, corrected for differential sequencing depth.

1.6.2 Individual Transcript Proportions

From the definition of of p_{ij}^f and Equation Equation (3): $p_{ij}^f = \alpha_j^f \frac{\mathbb{E}Y_{ij}^f}{\mathbb{E}Y_{ij}}$. To avoid numerical instabilities from the Gaussian approximation (which can produce negative values leading to $\mathbb{E}Y_{ij}^f > EY_{ij}$), we estimate:

$$p_{ij}^f = \frac{\alpha_j^f Y_{ij}^f}{\max\left(Y_{ij}, \sum_f \alpha_j^f Y_{ij}^f\right)}, \quad p_{ij}^u = 1 - \sum_f p_{ij}^f \tag{7}$$

. **Interpretation:** In theory, $\sum_f p_{ij}^f \leq 1$ always. However, because we approximate count data with Gaussian noise (which can be negative), some transcripts may have $\sum_f \alpha_j^f Y_{ij}^f > Y_{ij}$, which would yield invalid proportions >1. The max() function prevents this by using the larger value as denominator, effectively setting $p_{ij}^u = 0$ for such transcripts. This is a pragmatic solution to maintain valid proportions while using the computationally efficient Gaussian approximation.

1.7 Statistical Testing

1.7.1 Differential Proportion Analysis

To compare true relative abundances of individual transcripts across a fraction between two conditions, we use a binomial generalized linear model.‘ For conditions $\ell \in \{1, 2\}$:

$$\forall j \in \ell,\ Y_{ij}^f | \alpha_j^f, Y_{ij} \ \sim Bin(Y_{ij}, \alpha_j^f p_{i\ell})$$

We test:

- $H_0 : p_{i1}^f = p_{i2}^f$ (no change in fraction proportion between conditions)
- $H_1 : p_{i1}^f \neq p_{i2}^f$ (transcript shows differential fraction association)

P-values are adjusted for multiple testing using the Benjamini-Hochberg procedure.

Alternative methods: We also provide beta-binomial Wald test and logit-based test implementations as faster alternatives with potentially reduced statistical power.

Interpretation: This tests whether a transcript's distribution across fractions differs between biological conditions, controlling for the global fraction weights estimated by the model.

1.8 Summary

FracFixR solves the compositional challenge in fractionated RNA-seq through three key steps:

1. **Global fraction estimation**: Uses NNLS regression to estimate recovery ratios (α coefficients) that reveal true fraction weights, including unsequenced material
2. **Individual transcript correction**: Applies these global weights to calculate each transcript's true distribution across fractions
3. **Statistical inference**: Enables valid differential testing between conditions while accounting for compositional constraints

The framework is applicable to any RNA fractionation experiment (polysome profiling, subcellular localization, RNA-protein complex isolation, *etc.*) where both total and fractionated samples are sequenced.

2 FracFixR structure

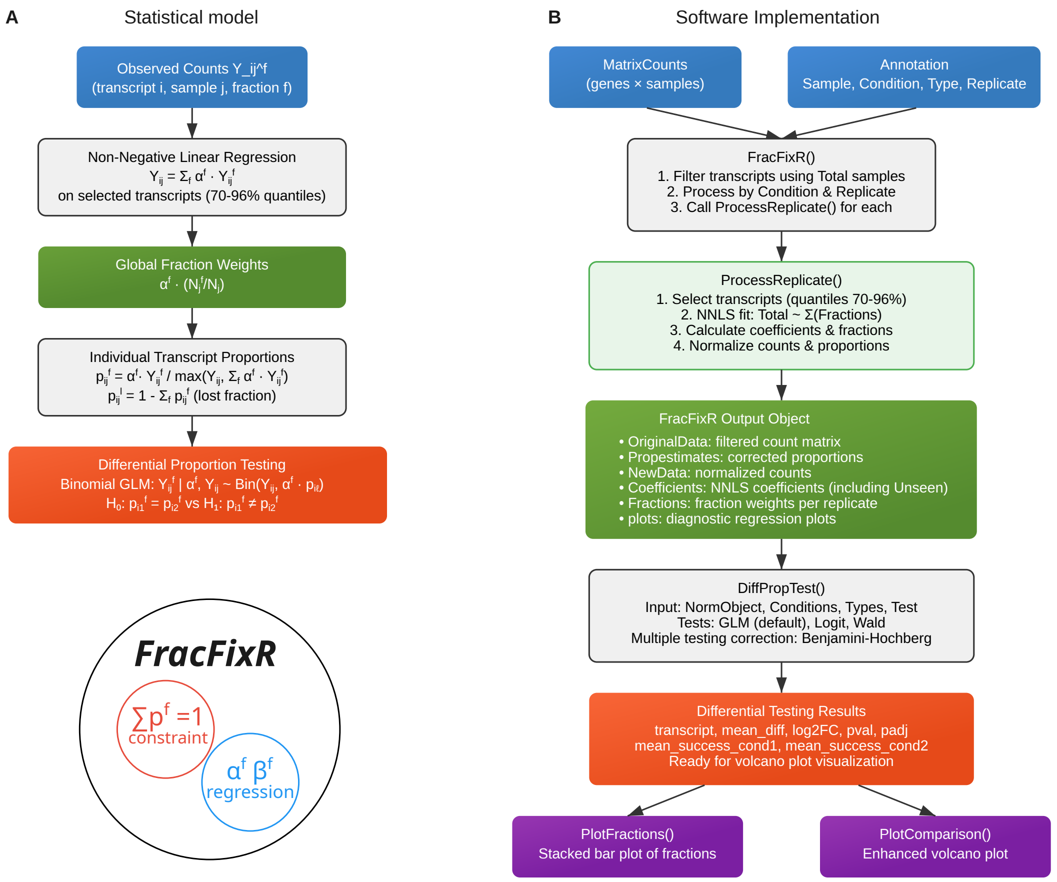

Fig. 1: `FracFixR` **statistical model and software implementation workflow.**

(**A**) Statistical model of `FracFixR`. The framework begins with observed counts Y_{ij}^{f} (reads for transcript i, sample j, fraction f) and uses non-negative linear regression (NNLS) on transcripts within 70-96% quantiles of total counts. This yields global fraction weights (α^{f}) representing each fraction's proportion in the original material. Individual transcript proportions (p_{ij}^{f}) are calculated from these weights, with the unseen fraction (p_{ij}^{u}) estimated as one minus the sum of observed fractions. Differential testing uses binomial GLM to compare transcript proportions between conditions. (**B**) Software implementation of `FracFixR`. `FracFixR` accepts MatrixCounts (genes by samples) and Annotation (Sample, Condition, Type, Replicate). The main function processes data by condition and replicate, calling ProcessReplicate() which: selects transcripts, fits NNLS regression, calculates fraction weights, and normalizes counts. Output includes: OriginalData, Propestimates (corrected proportions), NewData (normalized counts), Coefficients, Fractions, and diagnostic plots. DiffPropTest() performs differential analysis using GLM, Logit, or Wald tests with Benjamini-Hochberg correction. Visualization functions PlotFractions() and PlotComparison() generate stacked bar plots and volcano plots respectively. `FracFixR` handles multiple conditions/replicates, requires at least one "Total" sample per condition, automatically quantifies the unseen fraction, supports parallel processing, and works with any RNA fractionation protocol. Key notations: Y_{ij}^{f}: observed counts; alpha-f: fraction weights; p_{ij}^{f}: transcript proportions; p_{ij}^{u}: unseen fraction.

3 Synthetic and Real Data Generation

3.1 Fully synthetic data

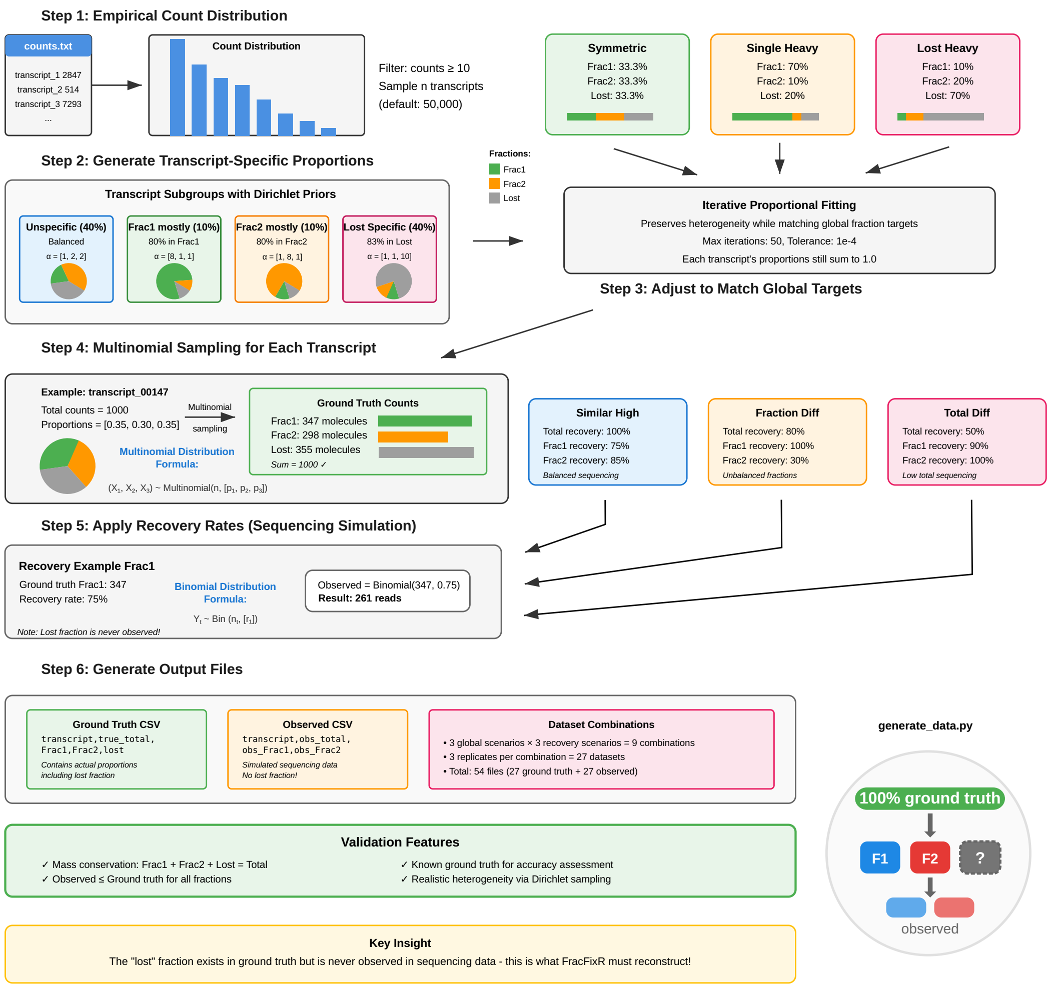

Fig. 2: **Synthetic data generation workflow for FracFixR validation.**

Step-wise process to generate synthetic RNA-seq datasets with known ground truth for validating `FracFixR` performance. Step 1 begins with empirical count distribution from real RNA-seq data, filtering transcripts with counts greater than or equal to 10 and sampling n transcripts (default 50,000). Step 2 assigns transcripts to four subgroups with distinct fractionation behaviors using different Dirichlet prior distributions: Group 1 (40% of transcripts) with symmetric distribution, Group 2 (10%) enriched in Fraction 1, Group 3 (10%) enriched in Fraction 2, and Group 4 (40%) with high lost fraction. The pie charts show typical fraction distributions for each group. Step 3 applies iterative proportional fitting to adjust individual transcript proportions while maintaining heterogeneity and matching global fraction targets. Step 4 implements three global scenarios representing different biological conditions: Symmetric (equal 33.3% distribution), Single Heavy (70% Frac1, 10% Frac2, 20% Lost), and Lost Heavy (10% Frac1, 20% Frac2, 70% Lost). Step 5 uses multinomial sampling to distribute each transcript's total counts across fractions according to its specific proportions, ensuring mass conservation. Step 6 simulates sequencing

by applying recovery rates through binomial sampling, testing three recovery scenarios: Similar High (balanced high recovery), Fraction Diff (unbalanced fraction recovery), and Total Diff (low total recovery with high fraction recovery). The example shows how ground truth counts are converted to observed reads. Step 7 generates output files: ground truth CSVs containing all fractions including lost material, and observed CSVs containing only sequenceable fractions, mimicking real experimental data where lost material cannot be observed. The workflow produces 54 files total (3 global scenarios $\times$ 3 recovery scenarios $\times$ 3 replicates $\times$ 2 file types), providing comprehensive test cases with known ground truth for assessing `FracFixR`'s ability to reconstruct unobserved fractions and correct for compositional biases.

3.2 Materials and Methods for model cell-lines

3.2.1 Cell lines and growth conditions

The B-ALL fusion-driven cell lines, REH and KOPN-8 cell lines were purchased from Leibniz Institute DSMZ, Germany. The non-B-ALL control cell line, GM12878 cell line was purchased from Coriell Institute for Medical Research, USA. All three cell lines were cultured under controlled conditions at 37 ℃ and 5% CO2. The cells were cultured in RPMI-1640 media (Sigma-Aldrich), supplemented with sodium bicarbonate and devoid of L-glutamine. Additionally, fetal bovine serum (FBS) was added to the RPMI-1640 media to a final concentration of 10 %. Cells were split in a 1:4 split and maintained at a cell density of 2 million at $\tilde{9}0$ % confluency.

3.2.2 Total (whole cell) RNA extraction

For whole cell transcriptome analysis, each cell line was cultured in RPMI-1640 as described above. The cell pellets were collected when cells attained a minimum cell density of 2×10^6 cells per ml of 50 ml media at a cell viability of at least 95 %. This ensured optimal cell viability and yield for all experimental procedures including downstream direct RNA sequencing and inhibitor based-polysome profiling. For the pellet collection, cells were pooled and spun down at 500 $\times$ g for 5 minutes. The supernatant was carefully discarded using a serological pipette, and the resulting cell pellet was washed with 1 mL of ice-cold phosphate-buffered saline (PBS). Following the PBS wash, cells were centrifuged again at 4°C for 5 minutes at 500 $\times$ g to ensure thorough pelleting, and the supernatant discarded. The PBS wash step was repeated after which the cell pellet was immediately processed further or stored at -80°C.

Total RNA was then isolated using the PureLink RNA Mini Kit (Thermo Fisher Scientific) following the manufacturer's protocol. Briefly, cells were homogenised and lysed in lysis buffer, and RNA was selectively bound to the provided RNA-binding columns. Contaminants were removed through a series of wash steps, and purified RNA was eluted in RNase-free water. The quality and quantity of isolated RNA were assessed using a Nanodrop spectrophotometer (Thermo Fisher Scientific). This method ensured the extraction of high-quality RNA suitable for downstream applications such as direct RNA sequencing.

3.2.3 Translatome capture using polysome profiling

Translational cocktail inhibitor treatment and cell collection

Translational inhibitor cocktail, comprising of tigecycline, anisomycin and emetine, was administered to cells to preserve the translatome containing polysome-bound mRNA fractions. These prevalently utilised translational inhibitors bind to the elongating ribosome

at distinct stages of the elongation cycle of protein synthesis. Emetine binds to the eukaryotic SSU (40S ribosomal subunit), while tigecycline binds to SSUs broadly, including the prokaryotic variant, and is also known to stall elongation, even within mitochondria. Anisomycin, on the other hand, binds to LSU (60S ribosomal subunit). This combined action of inhibitors was used to enhance elongation stalling efficiency compared to single drug interventions (reference). The inclusion of this inhibitor cocktail stabilises translational and polysomal complexes during gradient fraction separation in polysome profiling, ensuring sufficient polysomal yield and a more authentic profile for the downstream direct RNA sequencing.

The antibiotic cocktail was prepared with Tigecycline (100 µL) added to a final concentration of 100 µg/mL, Emetine (10 µL) added to a final concentration of 20 µg/mL, and Anisomycin (133 µL) dissolved to a final concentration of 1 mM for every 20 mL of cells suspended in RPMI-1640 media. Following sequential addition of the premix to the wells and gentle pipetting, the cells were incubated until the 3-minute mark. Subsequently, the cells were pooled and centrifuged at 500 × g for 5 minutes. The resulting pellet was washed in 1 mL of 1× PBS and centrifuged again at 500 × g for 5 minutes at 4°C. The cell pellet was aspirated and stored at -80°C for subsequent cell lysis and gradient fractionation.

Cell Lysis and extraction

Lysis buffer was prepared by combining HEPES-KOH (pH 7.6) to a final concentration of 25 mM, KCl to a final concentration of 50 mM, DTT to 4 mM, $MgCl_2$ to 5.1 mM, EDTA to 0.1 mM, Igepal CA-630 to 0.5%, glycerol to 5% (v/v), and emetine at 40 μg/mL. All components were added and combined at stored at –80 °C, while DTT and EDTA were added only upon immediate use of the lysis buffer. The cell pellet, from overnight freezing at -80°C, was defrosted in ice and promptly treated with 1:2.5 ratio of pellet to lysis buffer supplemented with 1 l of 40 U/µL RNAsin Plus (Promega). Subsequently, the cell suspension was vigorously pipette-mixed through a 200 µL tip against a tube wall to avoid the introduction of air and foam formation. Further homogenisation was achieved by passing the suspension through 27.5G and then subsequently 31G syringes for up to 8-10 times each. Following this, the fully homogenised lysate was then centrifuged at 12,000 × g for 10 minutes at 4°C. The top layer containing the clarified lysate was collected avoiding cell debris material in the pellet, and stored at -80°C. Quality assessment of the lysate was performed by measuring optical density (OD) against that of the lysis buffer using a Nanodrop spectrophotometer.

Preparation of sucrose gradients

Light Sucrose (LS) Gradient Buffer was prepared by combining 25 mM HEPES-KOH (pH 7.6, 25°C), KCl to a final concentration of 100 mM, $MgCl_2$ to 15 mM, EDTA to 0.1 mM, and a final concentration of 15% (w/v) sucrose. Heavy Sucrose (HS) gradient buffer was formulated with the same components as the LS Gradient Buffer, but sucrose concentration was elevated to 45% (w/v). For both LS and HS gradient buffers, emetine was added to a final concentration of 10 g/mL.

Sucrose buffer premixes were created by sequentially combining varying proportions of sucrose buffers and freshly added DTT to a final concentration of 10 mM. Each premix was stored on ice to preserve its reducing properties. Subsequently, the premixes were cast into Beckman-Coulter thin wall polypropylene tubes using an eppendorf dispenser preset to 2.5 ml. The casting process involved sequentially dispensing 2.5 ml of each premix into the tubes, starting from the highest sucrose concentration (45 %) sequentially to

the lowest (15 %). The solution was layered into four distinct sections, each containing sucrose concentrations of 15 %, 25 %, 35 %, and 45 %, with each layer comprising 2.5 ml. After casting each layer, the tubes were promptly placed on dry ice for incremental freezing. This controlled freezing process ensured the formation of stable sucrose gradients within the tubes upon melting and reaching diffusional equilibration. The gradients were promptly stored at -80 °C. Before use, the tubes were transferred to a cold room to allow gradual thawing overnight prior to ultracentrifugation and gradient fractionation.

Gradient fractionation

Cell lysates were obtained after freezing at –80 °C freezer and were thawed on ice. Prior to the ultracentrifugation spin, each sucrose gradient centrifuge tube mass was measured and adjusted to ensure uniformity. Subsequently, a predetermined volume of cell lysate equivalent to an OD600-concentration of 50 OD units, was gently added to the top of each gradient, ensuring the pipette tip was submerged just beneath the surface of the 15 % sucrose layer and lysate was dispensed slowly minimising bubble formation. The prepared tubes were then placed within a chilled ultracentrifugation rotor and centrifuged for 2 hours at 45,000 rpm at 4 °C.

Upon completion, the tubes were carefully removed and placed in the cold room. Following this, 60% sucrose was run through the gradient fractionator, ensuring the absence of any air bubbles within the tubes. To establish baseline readings, the instrument was calibrated using an empty gradient without any cell lysate preloaded as a blank to ensure a stable baseline of the gradient is reached. This procedure was iteratively repeated for all subsequent sample gradients containing the cell lysate after ultracentrifugation. Finally, fractions containing light polysome (monosome, disome and trisome) sample tubes, and heavy polysome sample tubes (tetrasome, pentasome and onwards) were collected and preserved on dry ice, while the remaining portions were discarded.

Total RNA purification from polysome-bound fractions

Following the fractionation of polysomes, the fractions were pooled and combined into light and heavy polysomal fractions. To do so equal proportions of monosomes, disomes, and trisomes were combined and designated as the light polysome fraction, while resolved fractions of equal proportion from tetrasomes and higher order polysomes were pooled and labeled as the heavy polysome fraction. For RNA purification, EDTA was added to a final concentration of 50 mM and SDS to a final concentration of 2 % were added to each tube, followed by incubation at 55 °C for 5 minutes until the solution clarified. Subsequently, SPRI beads were resuspended, vortexed, and added to the sample at a 1:1 ratio of bead suspension to sample. After thorough mixing, the solution was incubated at room temperature for 5 minutes in a magnetic rack. The supernatant was discarded following placement of the tube on a magnetic rack, and the beads were washed with 1 mL of 80% ethanol in deionized water. Following careful removal of the wash solution, the beads were dried on the magnet for 2 minutes before being eluted with 24 L of deionized water. Subsequently, the absorbance profile and purity values of the purified RNA were assessed using a Nanodrop spectrophotometer. Finally, the sample was frozen at -80°C for storage until further downstream experiments, such as sequencing.

3.2.4 Nanopore direct RNA sequencing

RNA library preparation

All RNA libraries for direct RNA sequencing (DRS) were prepared according to the manufacturer's protocol provided by Oxford Nanopore Technologies (ONT), with minor deviations for optimisation purposes. Initially, the RNA sample underwent sequential mixing with T4 DNA Ligase Buffer (Thermo Fisher Scientific), RT Adapter RTA, T4 DNA Ligase HC, and RNase In Plus for the ligation of RT Adaptor, followed by an incubation period as prescribed by ONT SQK002 protocol. Subsequently, reverse transcription was carried out by supplementing the mixture with 10 mM dNTPs, 5× SS UV First Strand Buffer, 0.1 M DTT, Nuclease-free water, and Superscript IV RT as per manufacturer's instructions.

The resulting library underwent purification using Agencourt AMPure XP beads (Beckman), involving incubation, magnetic pelleting, ethanol washing, and resuspension steps. In the subsequent ligation step, the purified library was combined with RNA Adaptor RMX, T4 DNA Ligase HC, T4 DNA Ligase buffer, and molecular-grade water (mQ) to facilitate ligation with the library with a motor adaptor as per manufacturer's instructions. A final cleanup procedure utilising Agencourt AMPure XP beads was performed, followed by elution in elution buffer ELB. All incubation durations, concentrations, and temperatures were rigorously maintained in accordance with SQK002 protocol provided by ONT. The resulting RNA library was subsequently directly used for loading or preserved at 4°C prior to loading.

Flow cell loading and live sequencing setup

The library loading procedure adhered to the protocol outlined by ONT for the MinION Mk1B or PromethION platform. The flow cell was inserted under the clip of the MinION, ensuring proper thermal and electrical contact. Quality control of the flow cell was performed, and the priming port was opened to remove any air bubbles. A priming mix, comprising Flush Tether and Flush Buffer (Activated Flush Buffer), was prepared, degassed, and loaded into the flow cell as per manufacturer's instructions. Following this RNA library was prepared by mixing RNA Running Buffer, the ELB eluted RNA and molecular-grade water (mQ). Upon degassing, the library was gently loaded into the flow cell via the SpotON sample port in a dropwise manner. Finally, the priming port was closed, the SpotON sample port cover was replaced, and the MinION or PromethION was secured in place, following the recommended protocol guidelines. The DRS sequencing runs were conducted at room temperature ($\tilde{2}5$°C) up to a maximum duration of 72 hours. Live basecalling was disabled during the run and the file format for data acquisition was set as FAST5 files for downstream preprocessing steps and subsequent bioinformatic analysis.

3.3 Controlled DRS read mixing experiment

To assess the performance of our method under controlled yet realistic conditions, we constructed a series of semi-synthetic transcriptomic datasets using publicly available RNA-seq data. Three experimental settings were considered, each based on different combinations of cell line transcriptomes:

- GM - REH - KOPN-8: A mixture of full transcriptome data from the GM12878 (GM), REH, and KOPN-8 cell lines.

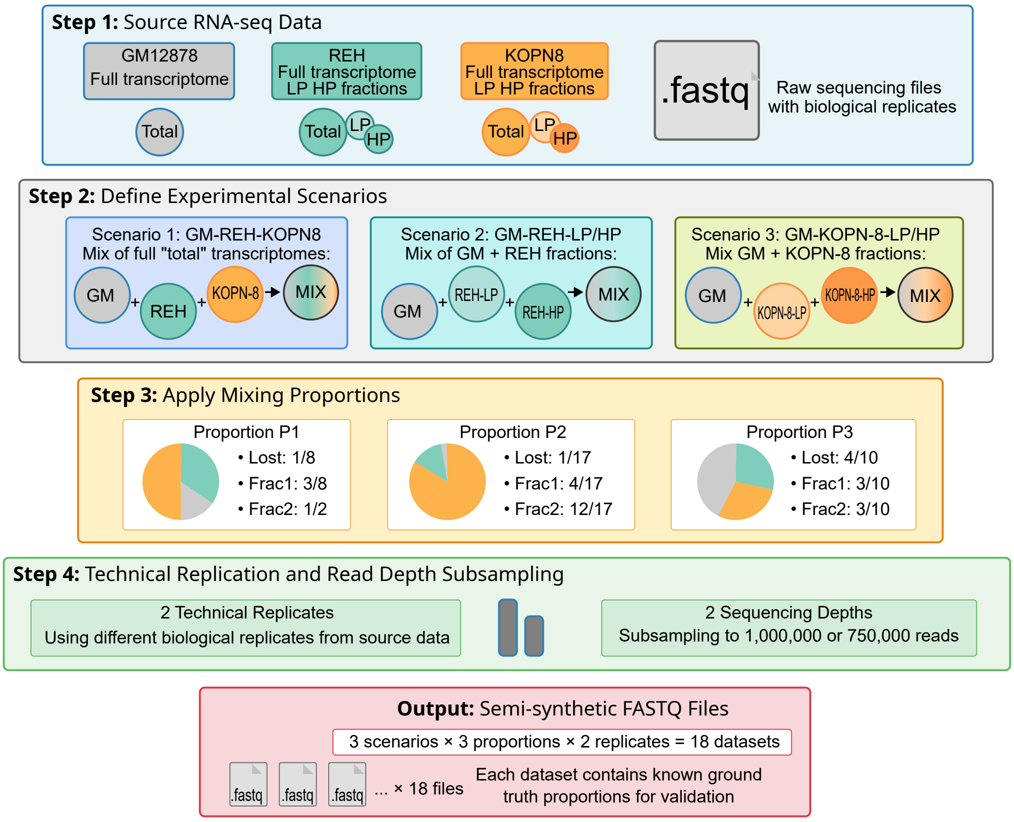

Fig. 3: **Controlled mixing data generation workflow for `FracFixR` validation.**

- GM - REH-LP/HP: A mixture of full transcriptome GM data with low-purity (LP) and high-purity (HP) REH samples.
- GM - KOPN-8-LP/HP: A mixture of full transcriptome GM data with LP and HP KOPN-8 samples.

For each of these scenarios, we constructed synthetic mixtures using three different mixing proportions:

- P1: 1/8 GM, 13/8 REH/KOPN-8, and 1/2 LP/HP
- P2: 1/17 GM, 4/17 REH/KOPN-8, and 12/17 LP/HP
- P3: 4/10 GM, 3/10 REH/KOPN-8, and 3/10 LP/HP

Each subcase (*i.e.*, combination of experimental scenario and mixing proportion) was replicated twice using different replicates from the original datasets, to simulate technical variability. For each replicate, we generated two sequencing depths by subsampling the raw FASTQ files to either 1,000,000 reads or 750,000 reads. Subsampling was performed using a custom in-house Python script, which randomly selected reads without replacement to match the desired read depth. This script is provided in the Supplementary Materials to ensure full reproducibility of the data generation process.

The resulting semi-synthetic datasets provide controlled, realistic mixtures of known transcriptomes, enabling robust benchmarking of normalization, quantification, and differential expression analysis pipelines.

4 FracFixR results on synthetic data

4.1 Fully synthetic data

Simulation Study Overview

To evaluate the performance of our method under controlled yet biologically realistic conditions, we designed a comprehensive simulation study based on a sequencing experiment involving a whole sample and two fractionated subsamples.

We considered three representative fractionation scenarios, each reflecting a distinct balance between the sequenced and unsequenced components:

1. **Balanced fractions** – the two sequenced fractions and the unobserved (lost) fraction contribute approximately equally to the total composition;

2. **Dominant sequenced fraction** – one of the sequenced fractions accounts for the majority of the total material, while the other and the lost fraction contribute less;

3. **Dominant lost fraction** – the unsequenced fraction represents the major component of the total sample, with the two sequenced fractions capturing only a minority of the total signal.

For each of these fractionation structures, we further examined three sequencing depth (recovery) scenarios designed to mimic realistic variability in experimental resource allocation:

1. **Deep sequencing across all samples**, providing high coverage for the total sample and both fractions;

2. **Limited depth for the total sample**, representing cases where most sequencing effort is allocated to the fractions rather than to the whole sample.

3. **Limited depth in one fraction**, simulating an imbalance in sequencing effort or sample quality between fractions;

Together, these nine combinations of fractionation and sequencing depth conditions span a broad range of realistic experimental settings, from ideal balanced designs to highly asymmetric or resource-limited situations. This design allows us to systematically assess the robustness and sensitivity of the proposed method to both compositional imbalance and uneven sequencing depth. It is summarized in Figure 4 with references to the results.

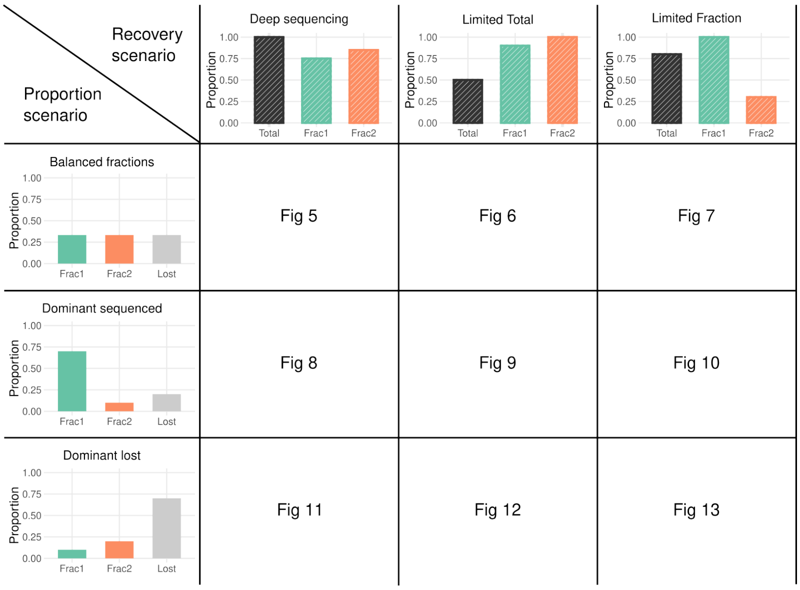

Fig. 4: **Roadmap of the synthetic data experiments, with references to result figures.** We considered three representative fractionation scenarios, each reflecting a distinct balance between the sequenced and unsequenced components, as well as three sequencing depth (recovery) scenarios designed to mimic realistic variability in experimental resource allocation.

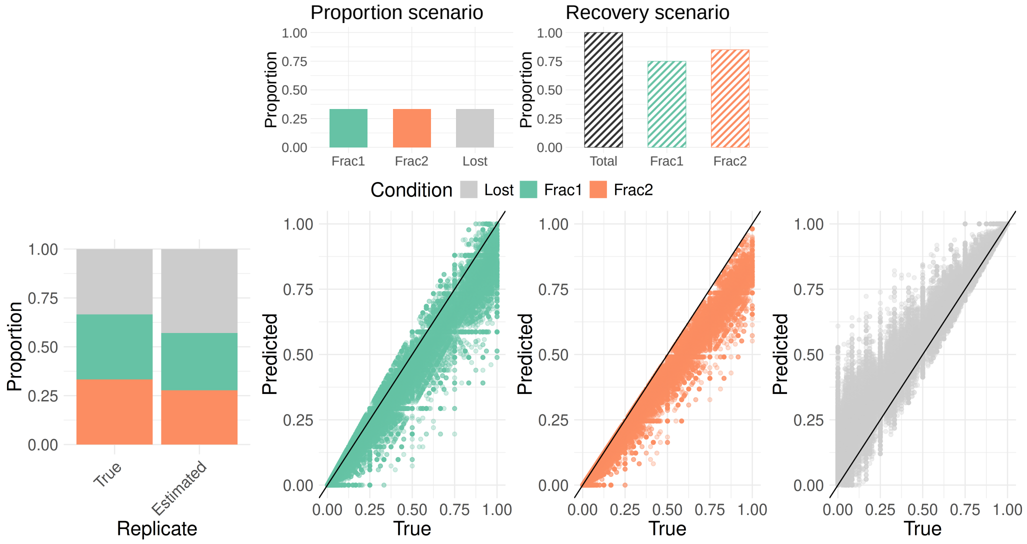

Fig. 5: `FracFixR` **validation on synthetic data symmetric proportion scenario, and high recovery rates in each sequencing experiment.** Top panels show ground truth fraction proportions (left) for each fraction (Frac1, Frac2, Lost) and recovery rates applied during sequencing simulation (right) for Total, Frac1, and Frac2 observations. Bottom panels display scatter plots comparing true versus `FracFixR`-estimated proportions for each fraction across all transcripts, with black diagonal lines indicating perfect recovery. Points represent individual transcripts, demonstrating `FracFixR`'s ability to accurately reconstruct both observed fractions and the unobserved lost fraction.

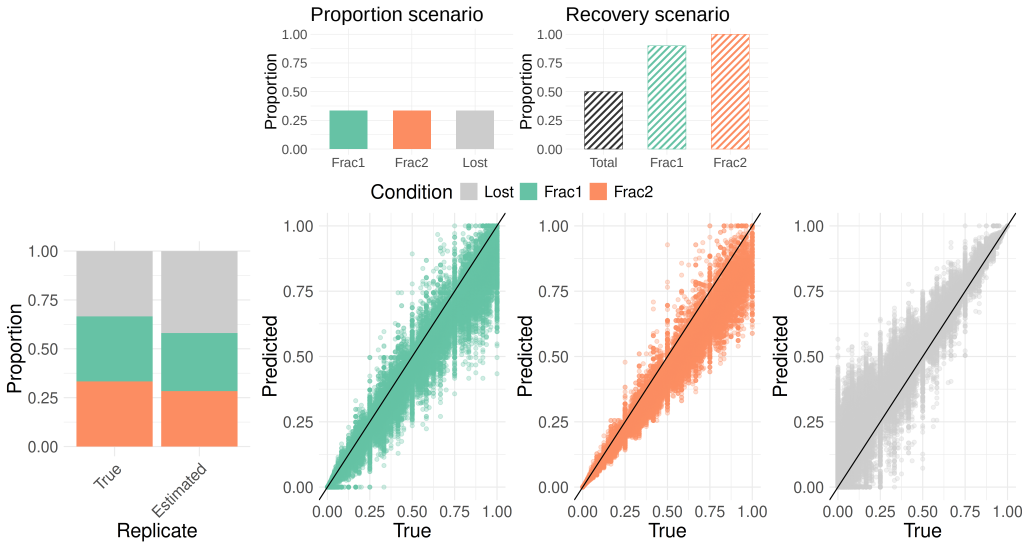

Fig. 6: `FracFixR` **validation on synthetic data with symmetric proportion scenario and low recovery rate for the total sequencing (Total Diff).** Experimental design and designations as in Supplementary Figure 5.

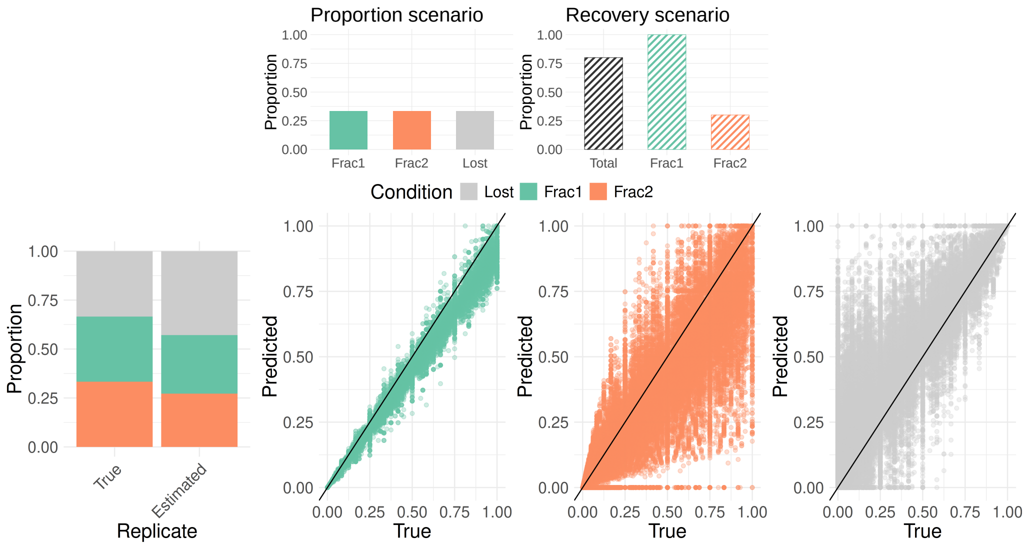

Fig. 7: `FracFixR` **validation on synthetic data with symmetric proportion scenario and low recovery rate for Frac2 sequencing (Fraction Diff).** Experimental design and designations as in Supplementary Figure 5.

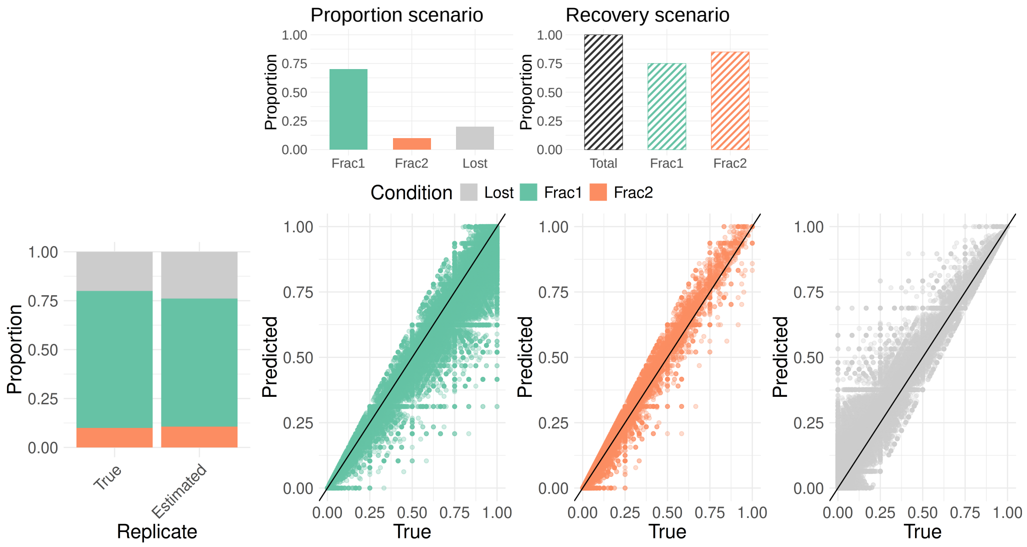

Fig. 8: **`FracFixR` validation on synthetic data with heavy proportion of Frac1 (scenario single heavy, with 60% Frac1 fraction), and high recovery rate (similar high scenario).** Experimental design and designations as in Supplementary Figure 5.

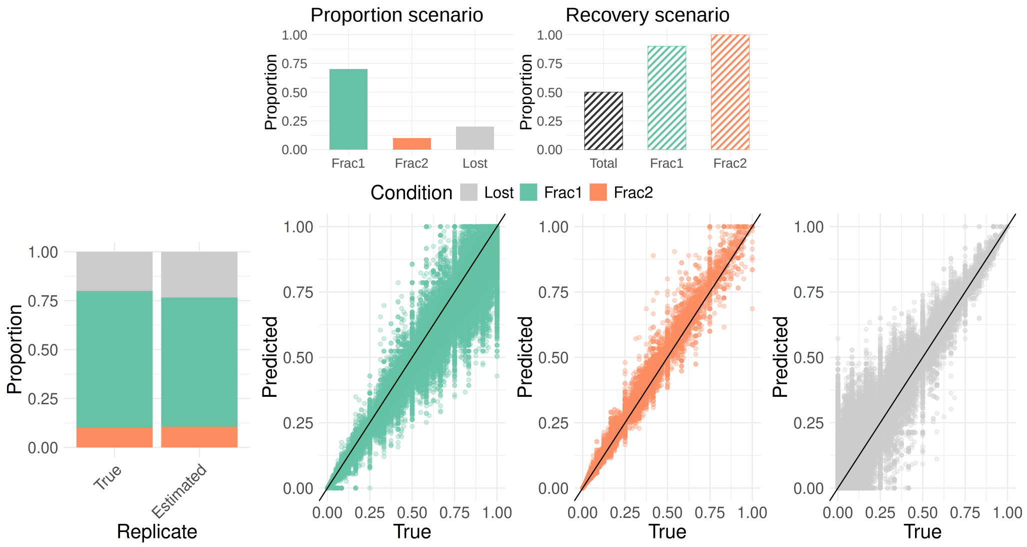

Fig. 9: **`FracFixR` validation on synthetic data with single heavy proportion scenario and total diff recovery scenario.** Experimental design and designations as in Supplementary Figure 5.

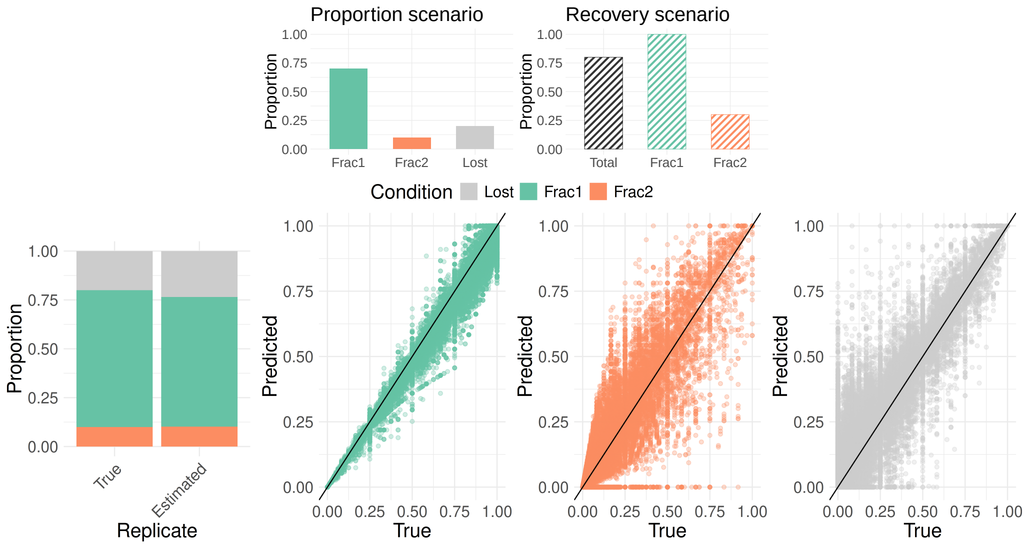

Fig. 10: `FracFixR` **validation on synthetic data with single heavy proportions under fraction diff recovery.** Experimental design and designations as in Supplementary Figure 5.

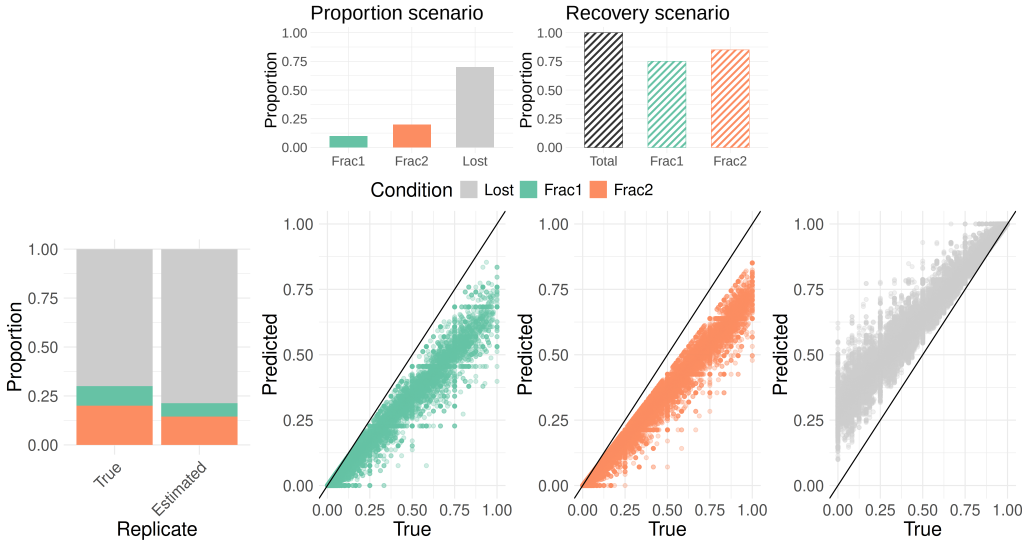

Fig. 11: `FracFixR` **validation on synthetic data with heavy proportion of the lost fraction (lost heavy) with similar high recovery scenario.** Experimental design and designations as in Supplementary Figure 5.

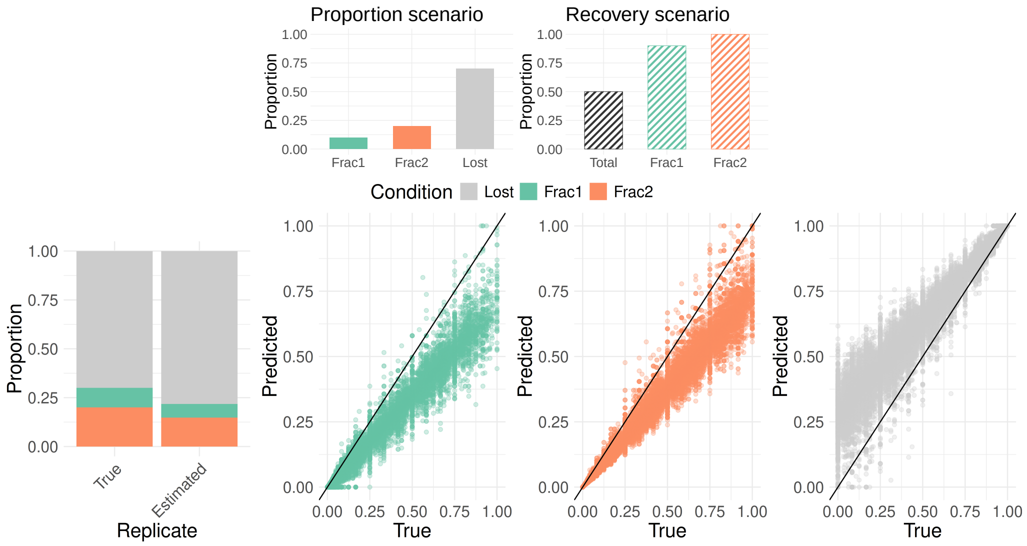

Fig. 12: `FracFixR` **validation on synthetic data with lost heavy proportions under total diff recovery.** Experimental design and designations as in Supplementary Figure 5.

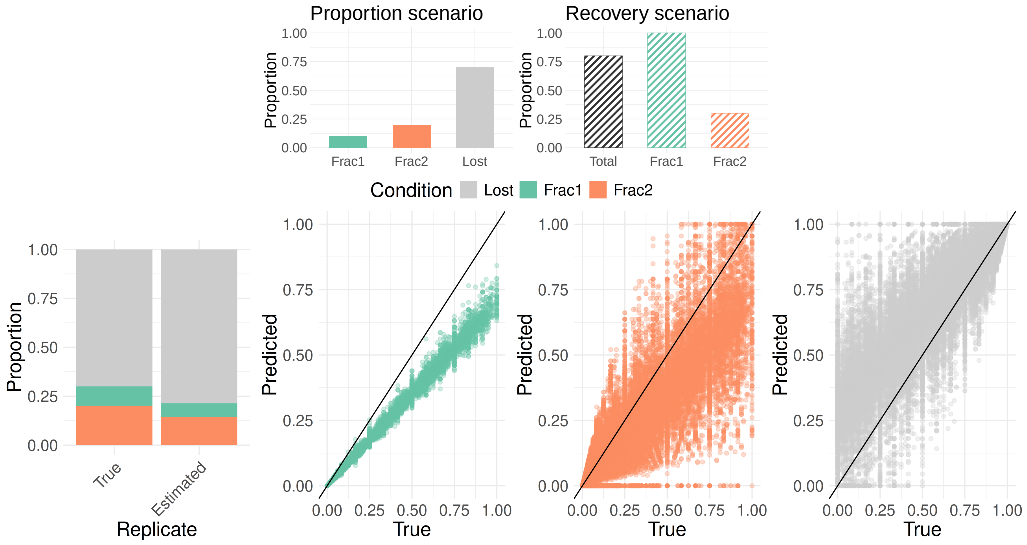

Fig. 13: `FracFixR` **validation on synthetic data with lost heavy proportions under fraction diff recovery.** Experimental design and designations as in Supplementary Figure 5.

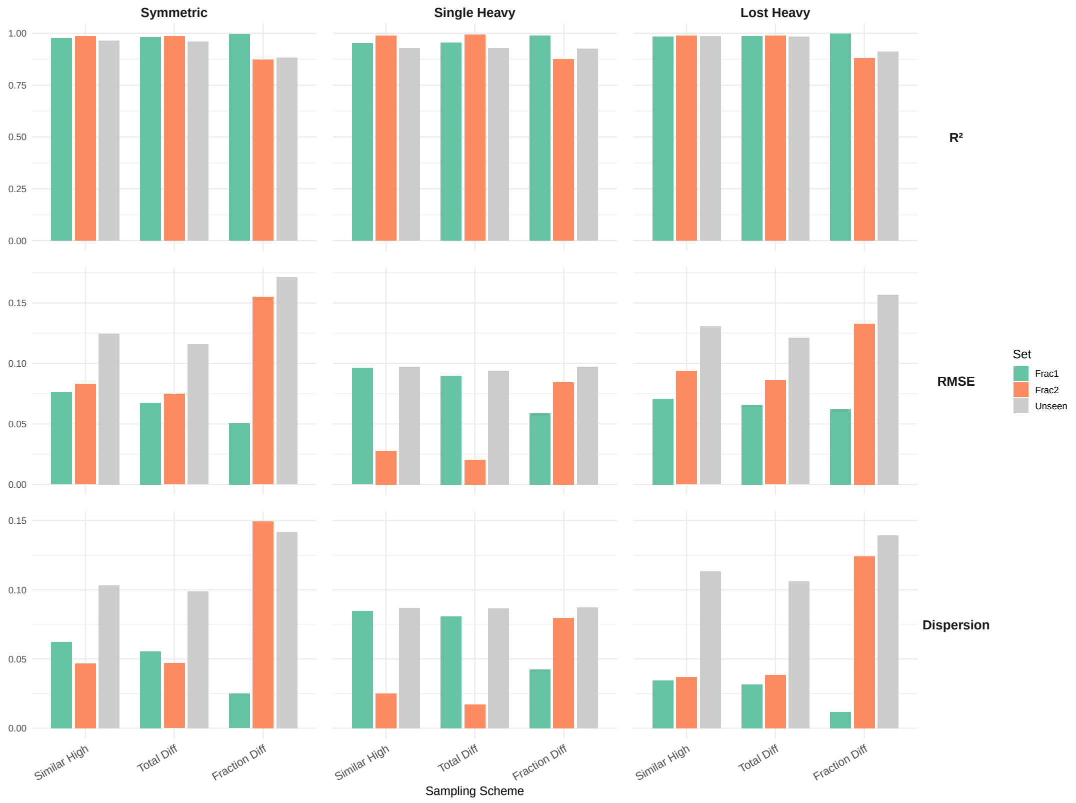

Fig. 14: **Comprehensive performance metrics of `FracFixR` across all synthetic validation scenarios.** Box plots show R^2 (coefficient of determination), RMSE (root mean square error), and dispersion values for each fraction (Frac1, Frac2, Unseen) across nine experimental conditions combining three proportion scenarios (Symmetric, Single Heavy, Lost Heavy) with three recovery schemes (Similar High, Total Diff, Fraction Diff). High R^2 values (>0.85) and low RMSE (<0.15) across all conditions demonstrate robust fraction weight reconstruction even under challenging recovery patterns.

4.2 Controlled real data

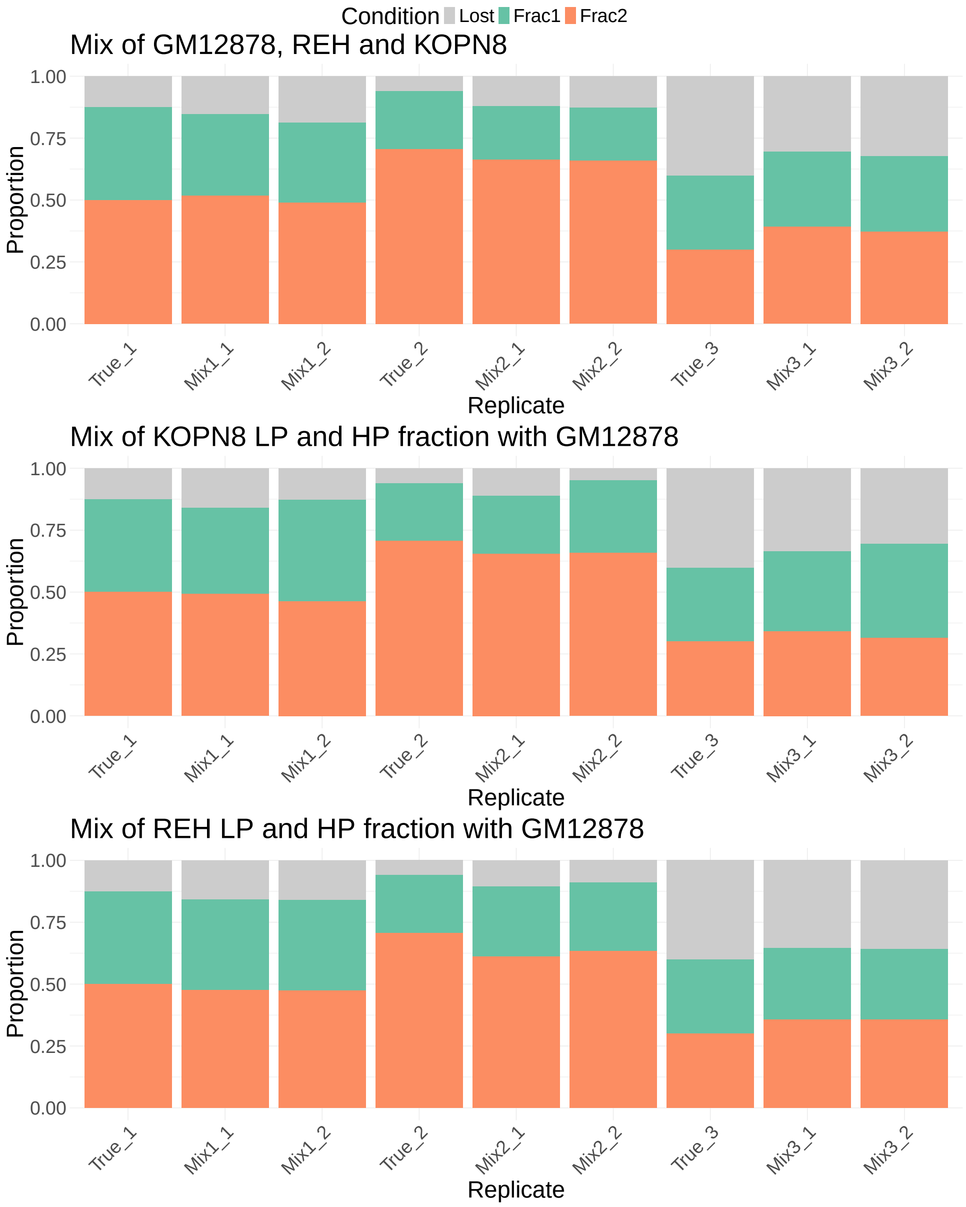

Fig. 15: `FracFixR` **reconstruction of fraction weights in controlled mixing experiments using real RNA-seq data.**

Stacked bar plots show the estimated proportions of lost (grey), Frac1 (orange), and Frac2 (green) fractions for three mixing scenarios: (top) equal mix of GM12878, REH, and KOPN-8 total cell lysates; (middle) mix of KOPN-8 polysomal fractions with GM12878 total lysate; (bottom) mix of REH polysomal fractions with GM12878 total lysate. True_N represents known mixing proportions, while Mix_N represents FracFixR estimates from observed data only in two different replicates, demonstrating accurate recovery of hidden GM12878 fraction as "lost" material.

5 FracFixR analysis of polysome profiling data

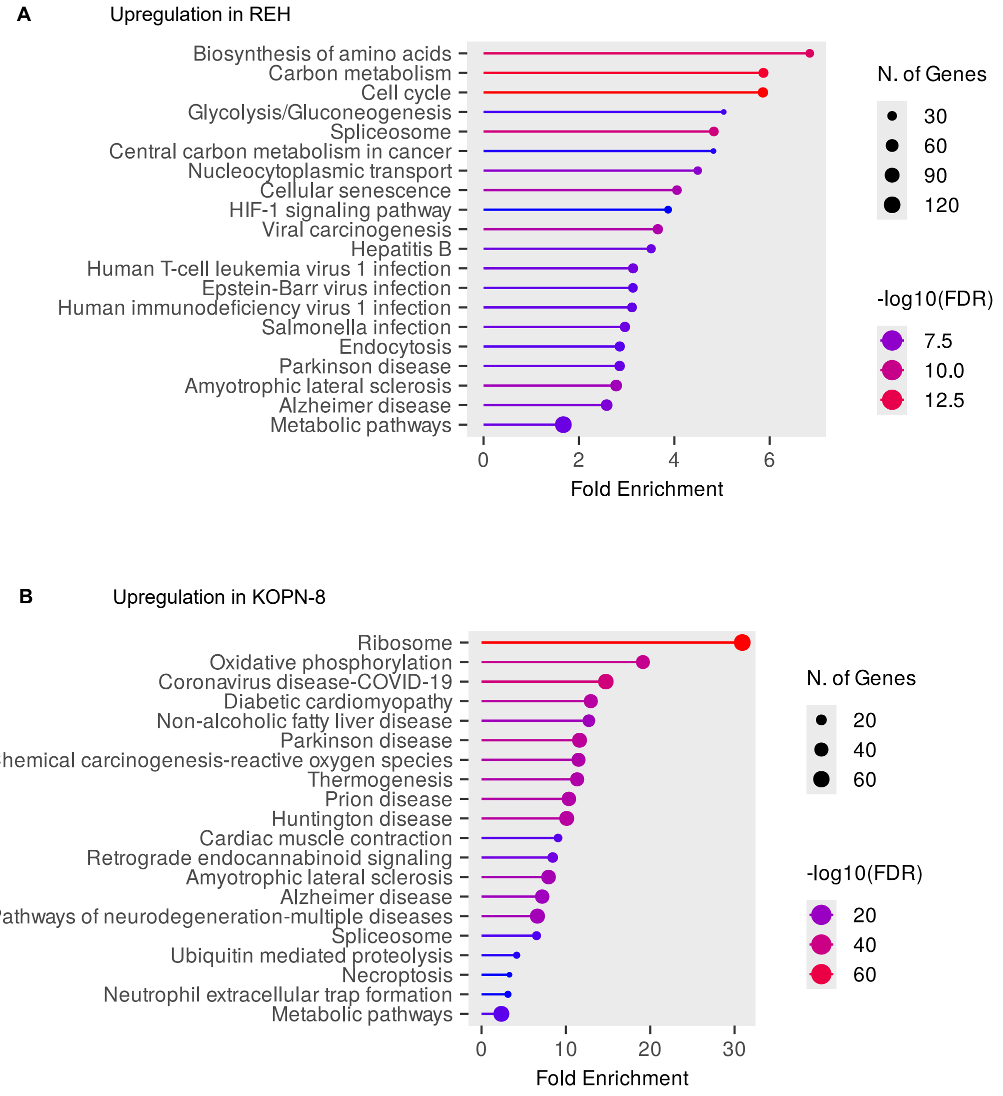

Fig. 16: **Pathway enrichment analysis reveals distinct translational programs between REH and KOPN-8 B-ALL cell lines.**

(**A**) Transcripts showing increased polysome association in REH cells are enriched for metabolic pathways, particularly biosynthesis of amino acids (fold enrichment 6), carbon metabolism, and glycolysis/gluconeogenesis. Notably, the spliceosome pathway shows significant enrichment, suggesting coordinated translational upregulation of RNA processing machinery. Central carbon metabolism in cancer and nucleocytoplasmic transport pathways are also enriched, indicating enhanced metabolic activity and cellular transport processes. (**B**) KOPN-8 cells show striking enrichment for ribosome components (fold enrichment 30), indicating substantial translational control of the protein synthesis machinery itself. This suggests a feed-forward mechanism where increased translation capacity is achieved through preferential translation of ribosomal proteins. Additional enrichment in oxidative phosphorylation and metabolic pathways indicates distinct metabolic programming compared to REH cells. The differential enrichment patterns reveal cell line-specific

translational control mechanisms: REH cells prioritize amino acid biosynthesis and RNA processing, while KOPN-8 cells enhance their translational capacity through ribosomal protein synthesis. Gene lists passing the significance and effect size cut-offs from the differential comparison engine of FracFixR (GLM) were further analyzed by ShinyGO 0.82 in the KEGG database. Dot size represents the number of genes in each pathway, color intensity indicates -log10(FDR), with darker colors representing more significant enrichment.
